# Modulation of Differentiation Processes in Murine Embryonic Stem Cells Exposed to Parabolic Flight-Induced Acute Hypergravity and Microgravity

**DOI:** 10.1089/scd.2017.0294

**Published:** 2018-06-15

**Authors:** Aviseka Acharya, Sonja Brungs, Margit Henry, Tamara Rotshteyn, Nirmala Singh Yaduvanshi, Lucia Wegener, Simon Jentzsch, Jürgen Hescheler, Ruth Hemmersbach, Helene Boeuf, Agapios Sachinidis

**Affiliations:** ^1^ Center for Molecular Medicine Cologne (CMMC), Institute of Neurophysiology, University of Cologne, Cologne, Germany.; ^2^ German Aerospace Center, Institute of Aerospace Medicine, Gravitational Biology, Cologne, Germany.; ^3^ INSERM-U1026, BioTis, University of Bordeaux, Bordeaux, France.

**Keywords:** microgravity, hypergravity, embryonic stem cells, differentiation, transcriptomics, parabolic flight

## Abstract

Embryonic developmental studies under microgravity conditions in space are very limited. To study the effects of short-term altered gravity on embryonic development processes, we exposed mouse embryonic stem cells (mESCs) to phases of hypergravity and microgravity and studied the differentiation potential of the cells using wide-genome microarray analysis. During the 64th European Space Agency's parabolic flight campaign, mESCs were exposed to 31 parabolas. Each parabola comprised phases lasting 22 s of hypergravity, microgravity, and a repeat of hypergravity. On different parabolas, RNA was isolated for microarray analysis. After exposure to 31 parabolas, mESCs (P31 mESCs) were further differentiated under normal gravity (1 *g*) conditions for 12 days, producing P31 12-day embryoid bodies (EBs). After analysis of the microarrays, the differentially expressed genes were analyzed using different bioinformatic tools to identify developmental and nondevelopmental biological processes affected by conditions on the parabolic flight experiment. Our results demonstrated that several genes belonging to GOs associated with cell cycle and proliferation were downregulated in undifferentiated mESCs exposed to gravity changes. However, several genes belonging to developmental processes, such as vasculature development, kidney development, skin development, and to the TGF-β signaling pathway, were upregulated. Interestingly, similar enriched and suppressed GOs were obtained in P31 12-day EBs compared with ground control 12-day EBs. Our results show that undifferentiated mESCs exposed to alternate hypergravity and microgravity phases expressed several genes associated with developmental/differentiation and cell cycle processes, suggesting a transition from the undifferentiated pluripotent to a more differentiated stage of mESCs.

## Introduction

Increasing attention is being given on the influence of gravity on physiology and developmental biology. It is hoped that progress in this emerging research field will help minimize health risks for astronauts, which face variable gravity conditions in space. Moreover, the vision of a future space settlement drives several scientific disciplines to investigate the influence of different gravity conditions on life and embryonic development under space conditions [1]. Embryonic development in space is associated with developmental defects in several animal species; these have been attributed to the effects of microgravity and radiation [2]. For example, experiments on the Russian space station Mir with the amphibian *Pleurodeles waltl* (Urodele) showed neural tube defects in developing embryos [3]. It was also observed that fertilized mice embryos did not develop under space conditions (Columbia, mission STS-80) [2].

To date, space conditions are known to have adverse effects on mammalian embryonic development. However, such studies are cost-intensive, very challenging to plan, as well as very rare, because of the restricted spaceflight opportunities. Therefore, most experiments investigating the effects of different gravity conditions have been performed under in vitro conditions using somatic cells in ground-based analog facilities that can simulate different gravity conditions to some extent.

Different types of ground-based facilities, such as the 2D clinostat, Random Positioning Machine, and Rotating Wall Vessel, are used to simulate microgravity as well as special centrifuges to apply hypergravity [4]. These facilities, several microgravity and hypergravity experiments with different mammalian cells have been performed, using the clinostat to simulate microgravity or special centrifuges to achieve hypergravity [5–7]. Although these studies worked with different cell types, they showed common alterations in cell morphology, the cytoskeleton, and cell function. Cytoskeletal changes are particularly gravity sensitive (see reviews in Refs. [2,5,8–11]).

To reach conditions closer to real microgravity (0 *g*) in the range of 0.01 *g*, more recently a parabolic flight approach has been applied. During parabolic flight experiments, an airplane repeats a parabolic flight path (normally 31 times), consisting of short periods (20–25 s) of 1.8 hypergravity, 0 *g* microgravity, and another 1.8 hypergravity phase. After completion of the first parabola, a 2-min 1 *g*-recovery period occurs before starting the second and subsequent parabolas. In addition, between the 15th and 16th parabola, a 1 *g*-recovery phase of 8 min occurs. However, there are some disadvantages of the parabolic flight approach, for example, 0 *g* phases are interspersed with 1.8 *g* hypergravity conditions (for review see Ref. [12]).

Nevertheless, in contrast to the clinostat, parabolic flight experiments facilitate investigation of the effects of acute (short period) and repeated exposure to hypergravity and microgravity on different cellular biological processes. It is well established that randomly differentiated embryonic stem cells (ESCs), isolated from the inner cell mass of a blastocyst, resemble early and late in vivo embryonic development [13–15]. Previously, we developed a so-called “pipette-based” methodology, suitable for mouse ESC (mESC) differentiation into embryoid bodies (EBs) within commercial plastic pipettes under 2D clinorotation [16]. In this previous study, mESCs were differentiated using the pipette-method for 3 days under normal 1 *g* conditions, in contrast to simulated long-term microgravity induced by 3 days of clinorotation.

In another series of experiments, the 3-day-old control 1 *g* EBs and the 3-day-old-clinorotated EBs were further differentiated under normal 1 *g* conditions for 7 days. Using extensive transcriptome studies, we demonstrated that the most prominent biological processes in differentiated mESCs affected by the simulated microgravity exposure for 3 days belong to cardiomyogenesis. Clearly, microgravity deregulated several genes belonging to the cytoskeleton and to MAP kinase and focal adhesion transduction. Given that nothing is known about the mechanical stress induced by alternate hypergravity and microgravity on the differentiation potential of mESCs, we investigated the effects of repeated short-term gravity alterations on their differentiation, induced by parabolic flight experiments.

## Materials and Methods

### CGR8 cell culture

mESCs CGR8 (ECACC No. 95011018) were cultured on gelatin (0.2%)-coated flasks in Glasgow's minimum essential medium (Life Technologies, Darmstadt, Germany), with 2 mmol/L glutamine (Life Technologies), 50 μmol/L *β*-mercaptoethanol (Life Technologies), 1,000 units/mL leukemia inhibitory factor (LIF) (Merck Chemicals, Darmstadt, Germany), penicillin (100 units/mL)/streptomycin (100 μg/mL), and 10% fetal bovine serum (GIBCO, Life Technologies). The composition of the differentiation media was Iscove's Modified Dulbecco's Medium (IMDM) (Life Technologies, Darmstadt, Germany), with 2 mmol/L glutamine (Life Technologies), 1× nonessential amino acid, 100 μmol/L *β*-mercaptoethanol (Life Technologies), penicillin (100 units/mL)/streptomycin (100 μg/mL), and 20% fetal bovine serum (GIBCO, Life Technologies). For the differentiation, bacteriological Petri dishes were used.

### Experimental design

**FIG. 1.**
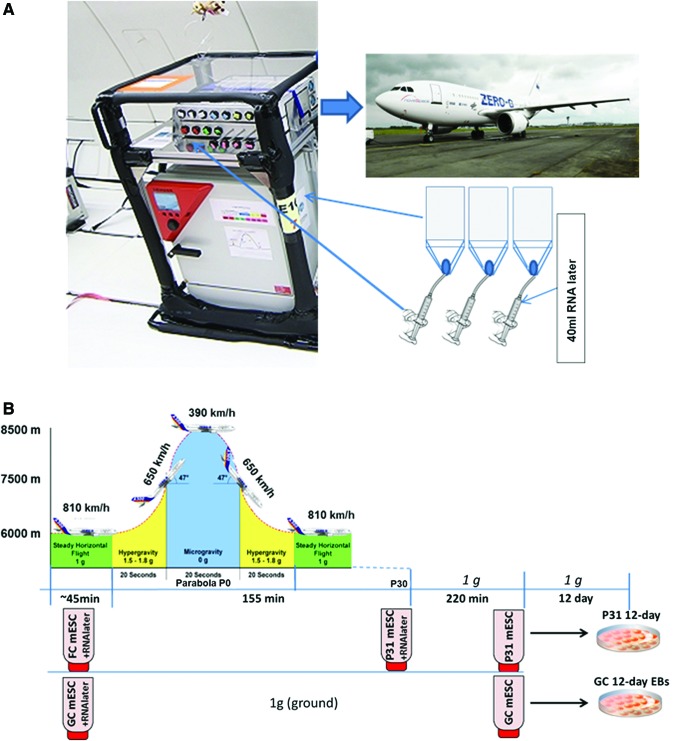
Experimental scheme for parabolic flight experiment. **(A)** An incubator was filled with T75 cell culture flasks, connected to syringes filled with RNA later in a fixation unit on *top* of the incubator. The syringes were manually operated from outside the incubator, injecting RNA later into the flasks at appropriate time points (before P0 and after P31). **(B)** Flight maneuver time points during the campaign and RNA later sample fixation points. Color images available online at www.liebertpub.com/scd

The parabolic flight experiments were performed by the Airbus A310 Zero-G (Novespace, Bordeaux Mérignac Airport, France) during the 64th ESA parabolic flight campaign. The experimental hardware consisted of a parabolic flight rack, including a normal cell culture incubator (CB 060, Binder, Tuttlingen, Germany) and a custom-made fixation unit for 16 different 50 mL-syringes (BD Plastipak, Heidelberg, Germany) (Fig. 1A). The syringes were connected to the cell culture flasks inside the incubator. A typical parabolic flight experiment comprises 31 parabolas, with alternating acceleration levels of regular gravity (1 *g*). Gravity conditions involve: (1) a hypergravity phase (1.8 *g*) of 20 s duration, (2) a hypogravity phase (∼0.01 *g*) of 22 s, and finally another hypergravity phase (1.8 *g*) of 20 s. The first maneuver of the Airbus A310 Zero-G to create a parabola always counts as the P0 parabola, while subsequent maneuvers are recorded as P1–P30. One campaign consists of three consecutive flight days, with 31 parabolas carried out on each day.

### CGR8 cell culture and sample fixation in parabolic flight experiment

To understand the effect of altered gravity on the differentiation processes of mESCs, cells were cultured in T75 cell culture flasks under standard cell culture conditions at 37°C and 5% CO_2_. In the culture flask, 10 mL of complete medium was maintained during the experiment. Equal numbers of replicate flasks (*n* = 3 for each of three flights) were prepared and kept on the ground (1 *g*) as control cells. One hour before take-off on flight days, 10 mL of fresh medium was exchanged into all flasks, before being segregated and transported to the 37°C preheated incubator (CB 060; Binder, Tuttlingen, Germany) on an experimental rack placed inside the Airbus 310, according to their various fixation time points (Fig. 1). Replicates of the flasks were kept inside the incubator (C60; Labotect, Göttingen, Germany) placed on the ground. Flasks were connected to the 50 mL syringes with flexible tubing via a three-way valve (except the flasks assigned for 12-day differentiation postflight). The syringes containing 45 mL fixative RNAlater^®^ (Life Technologies, Darmstadt, Germany) were administered by pushing a handle on the experimental hardware, causing the rubber stamp of the syringe to release the fixative into the cell culture flask (Fig. 1). The first fixation of mESCs was performed 30 s before P0, by manually pushing the handle to deliver 40 mL of RNAlater to the flask (flight control mESC [FC mESCs]) (Fig. 1B). At the same time, control cells on ground were fixed in the same manner, based on flight radar data (forming the ground control mESC [GC mESCs] samples) (Fig. 1B). Final samples were fixed after P31 (the last parabola, forming P31 mESC samples) (P31 mESCs). After the flight, the samples were immediately brought to the cell culture laboratory at (Inserm-U1026; BioTis University Bordeaux), washed with PBS (Life Technologies) twice, and the cell plate was collected in 1 mL TRIzol^®^ (Life Technologies), snap frozen and stored at −80°C for RNA isolation.

### Differentiation protocol

Both GC mESCs and mESCs exposed to the 31 parabolas (P31 mESCs) without fixation were further differentiated under ground 1 *g* conditions for 12 days, using the bacteriological Petri dish single-cell protocol to produce GC, FC, and P31 12-day EBs, respectively. In this case, the cells from the ground and parabolic flight experiment were trypsinized with Trypsin-EDTA (0.05%) (Life Technologies) and 2 × 10^4^ number of cells were seeded in differentiation medium (IMDM) in a bacteriological Petri dish and cultured at 37°C and 5% CO_2_ at 1 *g* for 12 days; medium was replaced every alternate day. On 12th day, differentiated medium was gently removed from the Petri dish and cells were fixed by adding 1 mL TRIzol and stored at −80°C for RNA isolation.

### RNA isolation and microarray experimental details

RNA isolation from the samples was performed following reported methods. In brief, total RNA was isolated using TRIzol and chloroform (Sigma, Steinheim, Germany) and purified with a miRNeasy Mini Kit (Qiagen, Hilden, Germany). Quantification was carried out using a NanoDrop (ND-1000; Thermo-Fisher, Langenselbold, Germany). For microarray labeling, 100 ng total RNA was taken as a starting material, and after amplification (Affymetrix^®^ standard protocol), 12.5 μg of amplified RNA was hybridized on Mouse Genome 430 version 2.0 arrays (Affymetrix, Santa Clara, CA) for 16 h at 45°C. The arrays were washed and stained in a Affymetrix Fluidics Station-450, according to the manufacturer's instructions. After staining, arrays were scanned with an Affymetrix Gene-Chip Scanner-3000-7G. Affymetrix GCOS software was used for quality control analysis.

### Statistical analysis

Statistical data analysis and visualization of microarray data were performed by uploading CEL files to the Transcriptome Analysis Console (Affymetrix). The gene intensity values were generated using Robust Multiarray Average background correction, quantile normalization, log_2_ transformation, and median-polished probe set summarization. The normalized genes were used for principal component (PC) analysis (PCA), while one-way ANOVA were used to generate differentially regulated transcripts with at least a 2-fold change (*P* ≤ 0.05). The online free software “Database for Annotation, Visualization, and Integrated Discovery” (DAVID) was used to determine functional annotation and Gene Ontology categories (GOs) of differentially expressed transcripts.

## Results

### Visualization of the parabolic flight-specific transcriptomes

**FIG. 2.**
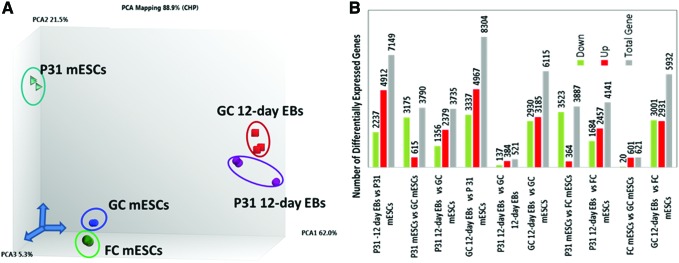
Analysis of the differentially expressed genes in undifferentiated and differentiated mESCs under variable gravity conditions. **(A)** The 3D-PCA carried out using TAC4.0 software (Affymetrix) based on the *limma* R/Bioconductor software package for integrative analysis of large scale gene expression data. Deregulated gene expression structure of transcriptome data sets was dimensionality-reduced and presented in the form of a 3D-PCA diagram. As expected, there were large differences between GC and FC mESCs, as well as GC 12-day and P31 12-day EBs. PCA1 illustrates a relatively large distance between GC and FC mESCs, and P31 mESCs. Small differences between GC 12-day EBs and P31 12-day EBs were observed, indicating lower numbers of differentially expressed genes in mESCs exposed to 31 parabolas and differentiated for 12 days under 1 *g* conditions. **(B)** Number of differentially expressed genes in undifferentiated and 12-day differentiated EBs under variable gravity conditions. The differentially expressed genes (<2-fold change in downregulated or >2-fold change in upregulated genes; *P* < 0.05) were identified by analyzing the CEL files using TAC4.0 and the *ebayes* (*limma*) gene expression analysis settings. mESCs, mouse embryonic stem cells. EB, embryoid body; GC and FC mESC, ground control and flight control mESC; PCA, Principle Component Analysis. Color images available online at www.liebertpub.com/scd

Visualization of the differences between the transcriptomes of the cell populations was performed using PCA, yielding the gene expression variability levels under different gravity conditions (Fig. 2A). As expected, the PC1 showed a pronounced gene expression variance of more than 50% between the undifferentiated GC mESCs and 12-day-differentiated mEBs (GC 12-day EBs), as well as between undifferentiated mESCs exposed to 31 parabolas (P31 mESCs) and those that underwent a further 12-day differentiation at 1 *g* (P31 12-day EBs). This demonstrates significant deregulation between the transcriptomes of the undifferentiated and differentiated 12-day EBs. Both PC2 and PC3 showed smaller gene expression variances of less than 10% and 3.5%, respectively. Transcriptome differences were observed between the GC and P31 mESCs (mainly in PC2), as well as between GC and P31 12-day EBs. Overall, the numbers of differentially expressed significant transcripts (*P-*value ≤0.05; fold change ≥ ± 2) are shown in Fig. 2B. The number of deregulated genes was very high in the differentiated GC and P31 12-day EBs versus their undifferentiated GC and P31 mESCs, respectively (6,115 and 7,149 genes). Interestingly, 3,887 genes were deregulated (364 upregulated, 3,523 downregulated) in P31 mESCs compared with the FC mESCs. In general, the number of differentially expressed genes in FC mESCs was relatively low (621-deregulated genes) compared with GC mESCs.

### Analysis of the genes affected in mESCs after 31 parabolas

Of particular interest was the identification and analysis of differentially expressed genes following exposure to 31 parabolas (P31 mESCs) compared with FC mESCs (Fig. 1B). Both cell populations were cultured in the presence of LIF. To identify biological processes and signal transduction pathways enriched or suppressed after exposure to 31 parabolas, the up- and downregulated genes were analyzed using tools in DAVID. The number of transcripts that were downregulated (3,175 genes) was significantly higher than upregulated ones (364 transcripts). Table 1 (for a complete list of GOs and genes belonging to each GO category see Supplementary Table S1; Supplementary Data are available online at www.liebertpub.com/scd) shows several GOs related to embryonic development and to development and differentiation of organ-specific cells that were significantly enriched in the P31 mESCs, despite being cultured in the presence of LIF and therefore under a pluripotent stage. We also found that KEGG signaling pathways, TGF-β signaling, and glycosaminoglycan degradation to be significantly enriched in P31 mESCs. Surprisingly, most of the deregulated genes were downregulated. DAVID analysis of these genes clearly indicated that cell cycle-related proliferative GOs and KEGG signaling pathways were repressed.

**Table 1.** Parent and Child Term GOs and KEGG Pathways Specifically Enriched into Mouse Embryonic Stem Cells After 31 Parabolas (P31 mESCs) in Comparison to Mouse Embryonic Stem Cells Before the Parabolas (FC mESCs)

*Term*	*Gene no.*	P	*% Coverage*
GO:0001568∼blood vessel development	18	0.01612	5.68
GO:0001649∼osteoblast differentiation	8	0.04935	2.21
GO:0001822∼kidney development	12	0.00569	3.79
GO:0001890∼placenta development	10	0.00128	3.15
GO:0001942∼hair follicle development	7	0.00426	0.95
GO:0001944∼vasculature development	18	0.02578	5.68
GO:0003007∼heart morphogenesis	9	0.04182	2.84
GO:0005783∼endoplasmic reticulum	51	2.25E-06	16.09
GO:0005789∼endoplasmic reticulum membrane	36	1.02E-06	11.36
GO:0006915∼apoptotic process	37	0.03643	11.67
GO:0007423∼sensory organ development	16	0.04056	5.05
GO:0009887∼organ morphogenesis	29	0.00177	9.15
GO:0009967∼positive regulation of signal transduction	29	0.04019	9.15
GO:0014031∼mesenchymal cell development	7	0.04559	2.21
GO:0021915∼neural tube development	8	0.02357	2.52
GO:0030097∼hemopoiesis	24	0.00769	7.57
GO:0030510∼regulation of BMP signaling pathway	5	0.0398	1.58
GO:0034660∼ncRNA metabolic process	14	0.01388	4.42
GO:0043588∼skin development	14	2.81E-04	4.42
GO:0045646∼regulation of erythrocyte differentiation	4	0.02459	1.26
GO:0045667∼regulation of osteoblast differentiation	7	0.01484	2.21
GO:0048513∼animal organ development	83	8.99E-06	26.18
GO:0048568∼embryonic organ development	20	1.65E-04	6.31
GO:0048598∼embryonic morphogenesis	17	0.03431	5.36
GO:0060538∼skeletal muscle organ development	8	0.03006	2.52
GO:0061458∼reproductive system development	17	0.00323	5.36
GO:0070059∼intrinsic apoptotic signaling pathway in response to endoplasmic reticulum stress	5	0.01161	1.58
GO:0072358∼cardiovascular system development	25	0.02809	7.89
GO:0097190∼apoptotic signaling pathway	16	0.02863	5.05
GO:2001233∼regulation of apoptotic signaling pathway	13	0.01253	4.10
GO:2001235∼positive regulation of apoptotic signaling pathway	7	0.04347	2.21
GO:2001236∼regulation of extrinsic apoptotic signaling pathway	7	0.03395	2.21
mmu00531:Glycosaminoglycan degradation	4	0.00181	1.26
mmu04350:TGF-beta signaling pathway	7	4.71E-04	2.21

In particular, 42 downregulated genes belonged to GO:0090068–positive regulation of cell cycle process, and 21 downregulated genes belonged to the GO:0000086–G2/M transition of mitotic cell cycle (Table 2). Therefore, we assume that mitotic processes were suppressed in P31 mESCs. The only other GO biological process (GO:0006915–apoptotic process) that was repressed was related to apoptosis (Table 2).

**Table 2.** Parent and Child GO Terms and KEGG Pathways Specifically Suppressed in Mouse Embryonic Stem Cells After 31 Parabolas (P31 mESCs) Compared with Mouse Embryonic Stem Cells Before the Parabolas (FC mESCs)

*Term*	*Count*	P	*% Coverage*
GO:0000070∼mitotic sister chromatid segregation	43	3.10E-14	2.39
GO:0000075∼cell cycle checkpoint	43	1.84E-09	2.39
GO:0000086∼G2/M transition of mitotic cell cycle	21	1.35E-05	1.17
GO:0000281∼mitotic cytokinesis	10	0.001976	0.56
GO:0005634∼nucleus	899	1.04E-52	49.94
GO:0005643∼nuclear pore	15	0.0026653	0.83
GO:0005730∼nucleolus	158	5.92E-17	8.78
GO:0006396∼RNA processing	151	1.22E-23	8.39
GO:0006406∼mRNA export from nucleus	14	9.51E-05	0.78
GO:0006915∼apoptotic process	193	1.41E-04	10.72
GO:0007094∼mitotic spindle assembly checkpoint	10	8.54E-05	0.56
GO:0007346∼regulation of mitotic cell cycle	77	1.27E-10	4.28
GO:0008631∼intrinsic apoptotic signaling pathway in response to oxidative stress	8	0.0476467	0.44
GO:0010468∼regulation of gene expression	570	2.04E-37	31.67
GO:0016571∼histone methylation	28	6.89E-06	1.56
GO:0032007∼negative regulation of TOR signaling	8	0.0247236	0.44
GO:0032774∼RNA biosynthetic process	480	6.73E-31	26.67
GO:0043069∼negative regulation of programmed cell death	110	5.44E-04	6.11
GO:0043484∼regulation of RNA splicing	34	5.22E-11	1.89
GO:0044770∼cell cycle phase transition	62	3.66E-08	3.44
GO:0051028∼mRNA transport	33	1.12E-10	1.83
GO:0051225∼spindle assembly	23	1.75E-06	1.28
GO:0090068∼positive regulation of cell cycle process	42	1.73E-07	2.33
GO:0097193∼intrinsic apoptotic signaling pathway	37	0.0057664	2.07
GO:1901991∼negative regulation of mitotic cell cycle phase transition	28	1.88E-06	1.56
GO:1902036∼regulation of hematopoietic stem cell differentiation	4	0.0161565	0.22
GO:1902749∼regulation of cell cycle G2/M phase transition	17	1.56E-04	0.94
GO:1903047∼mitotic cell cycle process	142	2.74E-23	7.89
GO:2001020∼regulation of response to DNA damage stimulus	29	1.45E-04	1.61
GO:2001141∼regulation of RNA biosynthetic process	468	1.69E-30	26.00
mmu03013:RNA transport	36	1.24E-08	2.00
mmu03015:mRNA surveillance pathway	24	2.39E-07	1.33
mmu04010:MAPK signaling pathway	29	0.0166413	1.61
mmu04110:Cell cycle	24	2.47E-05	1.33
mmu04115:p53 signaling pathway	12	0.0084450	0.67
mmu04910:Insulin signaling pathway	18	0.0251697	1.00
mmu04915:Estrogen signaling pathway	14	0.0247794	0.78
mmu04922:Glucagon signaling pathway	14	0.028742	0.78

**FIG. 3.**
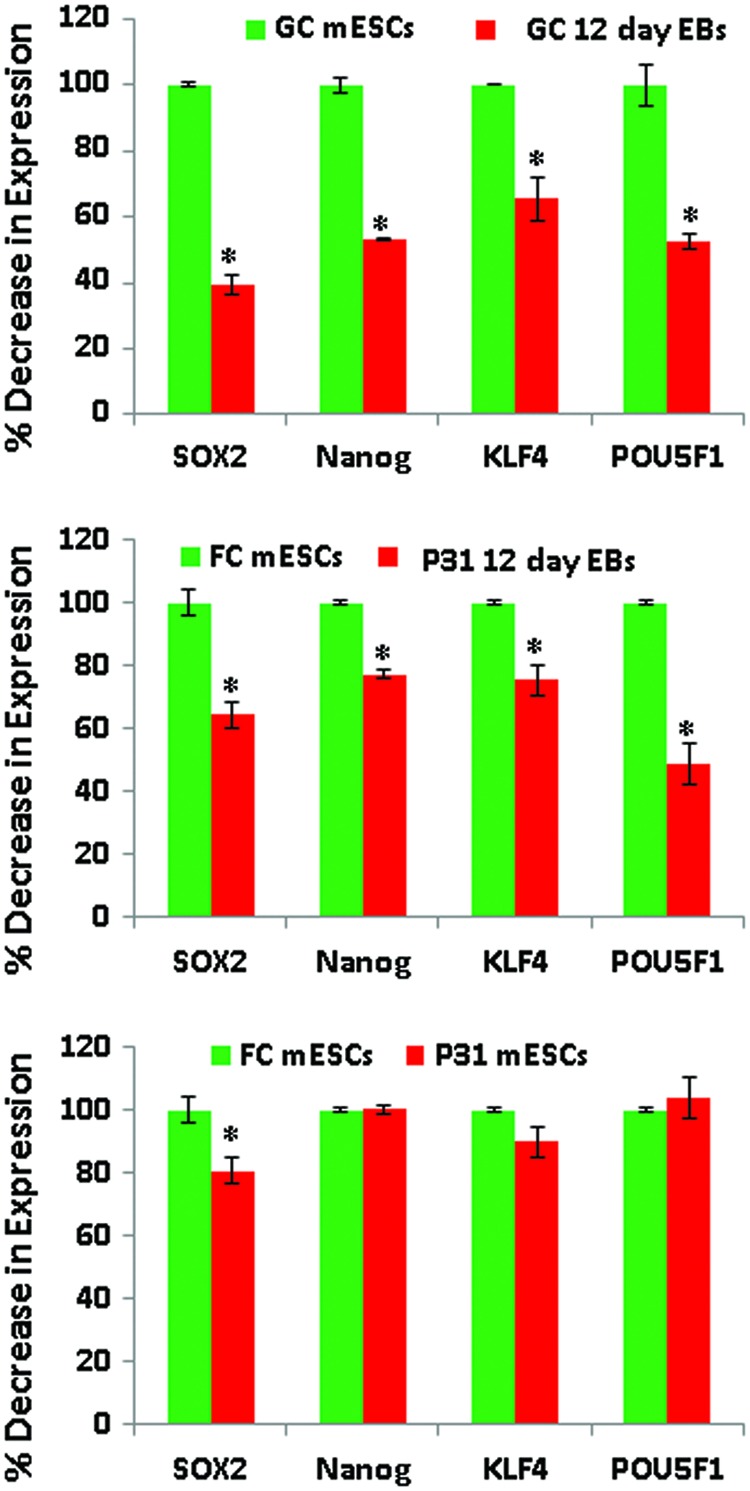
Expression of the pluripotent factors in GC mESCs, GC 12-day EBs, FC mESCs, P31 12-day EBs, and in FC mESCs versus P31 mESCs (*n* = 3; microarray expression values derived from three independent parabolic flights, executed over 3 days; *P* < 0.05 for mESCs vs. 12-day EBs). Color images available online at www.liebertpub.com/scd

Figure 3 shows the expression levels of the pluripotency promoting factors, such as *Pou5f1*, *Sox2*, *Nanog*, and *Klf4*, in GC 12-day EBs compared to GC mESCs, as well as P31 12-day EBs compared to P31 mESCs. The expression levels of all pluripotent factors were significantly lower in GC and P31 12-day EBs, because these factors are normally downregulated during differentiation of mESCs. However, except for Pou5f1, which had very similar levels in both GC and P31 12-day EBs, the expression levels of the other pluripotent factors were higher in P31 12-day EBs. The expression levels of all four pluripotent factors in P31 mESCs were similar to the levels in FC mESCs (except for *Sox2*) (Fig. 3), although the P31 mESCs expressed several genes associated with developmental processes.

### Analysis of the deregulated genes affected after differentiation of mESCs exposed to 31 parabolas

To identify genes affected by differentiation of mESCs exposed to the 31 parabolas with additional 12-day differentiation, we compared the differentially expressed genes between P31 12-day EBs and FC 12-day EBs (Fig. 1B). Comparison identified 551 deregulated genes in P31 12-day EBs; among them, 384 were upregulated and 137 downregulated. DAVID analysis of the upregulated genes showed enriched GOs associated with different developmental processes, such as neurogenesis, gland development, fat development, spermatogenesis, and other processes (Table 3). GOs that were specifically suppressed in P31 12-day EBs compared to FC 12-day EBs were associated with heart development and with the MAPK cascade, including both ERK1 and ERK2 cascades.

**Table 3.** GOs Specifically Enriched in Mouse Embryonic Stem Cells Exposed to 31 Parabolas and Differentiated for 12 Days Under 1-*g* Conditions (P31 12-Day Embryoid Bodies) Versus Flight Control 12-Day Embryoid Bodies (FC 12-Day Embryoid Bodies)

*Term*	*Count*	P	*% Coverage*
GO:0010468∼regulation of gene expression	92	2.49E-09	31.51
GO:0048468∼cell development	61	2.88E-09	20.89
GO:0016070∼RNA metabolic process	90	2.88E-08	30.82
GO:0048732∼gland development	23	1.39E-07	7.88
GO:0022008∼neurogenesis	41	1.50E-05	14.04
GO:0010628∼positive regulation of gene expression	40	8.63E-05	13.70
GO:0030182∼neuron differentiation	33	3.07E-04	11.30
GO:0006915∼apoptotic process	39	4.16E-04	13.36
GO:0043067∼regulation of programmed cell death	34	6.19E-04	11.64
GO:0016358∼dendrite development	11	7.19E-04	3.77
GO:0061458∼reproductive system development	16	0.001131047	5.48
GO:0045444∼fat cell differentiation	10	0.001152154	3.42
GO:0004672∼protein kinase activity	18	0.001432929	6.16
GO:0001825∼blastocyst formation	5	0.001822467	1.71
GO:0060548∼negative regulation of cell death	25	0.002020882	8.56
GO:0030879∼mammary gland development	8	0.00226657	2.74
GO:0007281∼germ cell development	12	0.002593612	4.11
GO:0061053∼somite development	6	0.005103724	2.05
GO:0001501∼skeletal system development	14	0.008336702	4.79
GO:0008406∼gonad development	9	0.012011842	3.08
GO:0008631∼intrinsic apoptotic signaling pathway in response to oxidative stress	4	0.012651921	1.37
GO:0007283∼spermatogenesis	15	0.013400423	5.14
GO:0060541∼respiratory system development	9	0.015552787	3.08
GO:0009952∼anterior/posterior pattern specification	8	0.017554488	2.74
GO:0072074∼kidney mesenchyme development	3	0.019887064	1.03
GO:0001568∼blood vessel development	15	0.025574845	5.14
GO:0007179∼transforming growth factor beta receptor signaling pathway	6	0.029268965	2.05
GO:0043586∼tongue development	3	0.034207515	1.03
GO:0001889∼liver development	6	0.042352299	2.05
GO:0009880∼embryonic pattern specification	4	0.044904479	1.37
GO:0048839∼inner ear development	7	0.045708507	2.40

## Discussion

To understand life under conditions in space, significant attention has been paid to space biology. In this context, evaluation of reproduction under conditions in space, applying in vivo and in vitro methodologies is of particular interest [2]. ESCs represent an optimal model to investigate developmental and differentiation processes under in vitro conditions [17]. Applying both, a simulated microgravity approach [18] and microarrays, we identified GOs and KEGG pathways in differentiated mESCs affected by microgravity exposure [17]. In addition, we could demonstrate that the most significantly downregulated genes in 3-day EBs exposed to simulated microgravity were cytoskeletal genes and genes involved in heart development [17]. Downregulation of cytoskeletal genes under microgravity conditions also has been reported in other cell types [19–22].

In the present study, we investigated the effects of alternate short periods of hyper- and hypogravity, occurring on a parabolic flight circuit, on the gene expression of mESCs cultured in the presence of LIF. Our results demonstrated that mESCs exposed to 31 parabolas significantly suppressed genes belonging to GO categories associated with cell proliferation, and in parallel, GOs associated with apoptosis. In addition, they had significantly enriched GOs associated with organ development, such as vasculature and kidney development, in parallel with GOs associated with apoptosis and the KEGG mmu04350:TGF-β signaling pathway.

Given that 110 downregulated genes (Table 2) belong to GO:0043069–negative regulation of programmed cell death, and 37 upregulated genes belong to GO:0006915–apoptotic process (Table 1), we assume that apoptosis was enhanced in the P31 mESCs. To date, there are no other studies investigating the effects of parabolic flight conditions on mESCs. Moreover, there are contradictory findings regarding the effects of microgravity on the cell cycle and cell proliferation. For example, mesenchymal stem cells cultured under simulated microgravity have a higher proliferative capacity, compared with cells cultured under normal conditions of 1 *g*, although they sustain their differentiation capacity [23]. Similar experiments on skeletal muscles showed a high proliferative capacity under simulated microgravity conditions [24].

Genome-wide expression profiling studies of cancer cells demonstrated that simulated microgravity induced a significant downregulation of several cell cycle genes, accompanied by inhibition of cell proliferation [25]. Likewise, it has been reported that microgravity simulated by clinorotation inhibits proliferation of bone marrow mesenchymal stem cells through inhibition of the cell cycle and enhanced apoptosis [26]. Similar results have been reported for the human gastric carcinoma cell line SGC-7901 and the human gastric normal cell line HFE-145, suggesting less proliferative capacity related to inhibition of the cell cycle under microgravity conditions [27]. It has also been reported that simulated microgravity inhibited proliferation and osteogenesis of rat bone marrow mesenchymal stem cells [28]. Based on these studies [26–28], we hypothesize that suppression of the GOs in mESCs associated with the cell cycle is related to their exposure to microgravity.

Our results also indicate enrichment of GOs in P31 mESCs associated with the developmental processes of various organs. Previous reports have suggested that simulated microgravity and weightlessness occurring during spaceflights can trigger stem cell and cancer stem cell differentiation [29–31]. There is increasing evidence that mechanical stress per se is sufficient to induce stem-cell transition, independently of hormones, toward differentiation to different somatic cells [31–33]. Therefore, we hypothesize that microgravity and/or hypergravity may induce mechanical stresses, promoting transition of undifferentiated mESCs to more differentiated mESCs. Interestingly, the expression levels of pluripotent factors, *Pou5f1*, *Nanog*, *Klf4*, and *Sox2*, in P31 mESCs were very similar to their levels in FC mESCs, although several genes associated with developmental processes were overexpressed in P31 mESCs. These findings suggest that changes in gravity-induced expression of genes associated with differentiation processes are unique, reflecting expression levels that are not regulated by classic pluripotent factors.

We also investigated the differentiation potential of mESCs exposed to 31 parabolas, followed by differentiation under 1 *g* conditions for 12 days (P31 12-day EBs), compared with differentiated GC 12-day EBs. No difference between the sizes of P31 12-day EBs and GC 12-day EBs was observed. Moreover, both the GC and the P31 12-day EBs had contractile areas related to differentiation of the cells to contactile cardiomyocytes (data not shown), suggesting differentiation occurred in both GC and P31 mESCs. However, we did not quantify cardiomyogenesis in GC and P31 12-day EBs. Several GOs involved in developmental processes were significantly enriched in the differentiated P31 12-day EBs, compared with the FC 12-day EBs (eg, GOs associated with blood vessels, the nervous system, spermatogenesis, skeletal development, and other categories; Table 3). Interestingly, among suppressed GOs in P31 12-day EBs, mainly GOs associated with heart development and with the MAP kinase cascade were identified (Table 4). These findings suggest that the developmental program initiated by the conditions experienced during the parabolic flight experiment in P31 mESCs, continued during further differentiation to P31 12-day EBs. We conclude that alternate changes of gravity, induced by the parabolic flight experiment, may enhance the differentiation potential of mESCs toward specific cell types, such as neurons or cell types of the cardiovascular system.

**Table 4.** GOs Specifically Suppressed in Mouse Embryonic Stem Cells Exposed to 31 Parabolas and Differentiated for 12 Days Under 1-*g* Conditions (P31 12-Day Embryoid Bodies) Versus Flight Control 12-Day Embryoid Bodies (FC 12-Day Embryoid Bodies)

*Term*	*Gene no.*	P	*% Coverage*
GO:0044267∼cellular protein metabolic process	27	3.12E-04	45.76
GO:0006915∼apoptotic process	15	6.28E-04	25.42
GO:0016310∼phosphorylation	14	0.007339259	23.73
GO:0005925∼focal adhesion	6	0.008186175	10.17
GO:0035556∼intracellular signal transduction	15	0.00961423	25.42
GO:0055002∼striated muscle cell development	4	0.012606213	6.78
GO:0055001∼muscle cell development	4	0.016495658	6.78
GO:0006508∼proteolysis	11	0.017068186	18.64
GO:0000165∼MAPK cascade	7	0.017651182	11.86
GO:0060047∼heart contraction	4	0.018011943	6.78
GO:0023014∼signal transduction by protein phosphorylation	7	0.018108933	11.86
GO:0055013∼cardiac muscle cell development	3	0.023377587	5.08
GO:0055006∼cardiac cell development	3	0.026239123	5.08
GO:0055017∼cardiac muscle tissue growth	3	0.02802094	5.08
GO:0060419∼heart growth	3	0.03236142	5.08
GO:0048738∼cardiac muscle tissue development	4	0.032510032	6.78
GO:0014706∼striated muscle tissue development	5	0.036231052	8.47
GO:0060548∼negative regulation of cell death	8	0.036790061	13.56
GO:0050684∼regulation of mRNA processing	3	0.038986259	5.08
GO:0070371∼ERK1 and ERK2 cascade	4	0.0458714	6.78

As previously described, differentiation of mESCs exposed to 3 days of simulated microgravity, followed by differentiation for seven additional days under 1 *g* conditions (10-day EBs) inhibited the process of cardiomyogenesis compared with control 10-day EBs [17]. Recently, it has been shown that 15-day EBs exposed to long-term microgravity (ISS shuttle conditions) induced an inhibition of mESC differentiation and retained stem cell self-renewal markers [34]. These authors reported that in cells recovered from microgravity unloaded EBs, and further cultured under 1 *g* earth conditions, differentiation into contractile cardiomyocytes occurred very readily. Differences between this report [34] and the present study can be explained by the differences in the experimental gravity conditions (long-term microgravity vs. short-term alternating hyper- and hypogravity conditions). Nevertheless, the main limitations of our experiment are that: (1) mESCs can only be exposed to different gravity conditions for a short time; and (2) neither exclusive effects of microgravity or hypergravity on the differentiation of mESCs can be investigated.

In conclusion, exposure of undifferentiated mESCs to alternate hyper- and hypogravity phases upregulated genes belonging to GOs and KEGG pathways associated with developmental processes, but downregulated several genes belonging to GOs associated with suppression of the cell cycle. Moreover, differentiation of mESCs for 12 days following exposure to hyper- and hypogravity phases resulted in upregulation of different organ developmental genes (Table 3) and downregulation of genes associated with heart muscle development (Table 4).

## Supplementary Material

Supplemental data
